# Incidence of Axillary Adenopathy in Breast Imaging After COVID-19 Vaccination

**DOI:** 10.1001/jamaoncol.2021.3127

**Published:** 2021-07-22

**Authors:** Kristin A. Robinson, Santo Maimone, Denise A. Gococo-Benore, Zhuo Li, Pooja P. Advani, Saranya Chumsri

**Affiliations:** 1 Department of Radiology, Mayo Clinic, Jacksonville, Florida; 2 Department of Internal Medicine, Mayo Clinic, Jacksonville, Florida; 3 Department of Quantitative Health Science, Mayo Clinic, Jacksonville, Florida; 4 Division of Hematology and Medical Oncology, Mayo Clinic, Jacksonville, Florida

## Abstract

This case series examines the incidence, timing, and characteristics of mammographic axillary adenopathy following COVID-19 vaccination.

Vaccine-induced adenopathy after COVID-19 vaccination in breast imaging has received significant media attention, with evolving literary correspondence on management. Patients’ self-report of axillary swelling following COVID-19 vaccination was reported as high as 16%.^1^ The National Comprehensive Cancer Network and Society of Breast Imaging recommended to consider scheduling screening breast imaging 4 to 6 weeks after the second COVID-19 vaccination dose when possible.^2^ However, the actual incidence, timing, and characteristics of mammographic axillary adenopathy following COVID-19 vaccination remain uncertain.

## Methods

Retrospective analysis was carried out assessing patients who received at least 1 injection of COVID-19 vaccine fewer than 90 days prior to either screening or diagnostic mammography at the Jacoby Center for Breast Health, Mayo Clinic, Florida, between January 15 and March 22, 2021. Information regarding COVID-19 vaccination and symptomatic adenopathy was inquired by technicians performing mammography and documented in the electronic medical record. Axillary adenopathy was assessed by interpreting radiologists and all adenopathy cases were re-reviewed. Wilcoxon rank-sum test and Fisher exact test were used to compare continuous and categorical variables, respectively. Multivariable logistic regression model was used to evaluate the association between days from vaccine and adenopathy. Receiver operating curve (ROC) analysis was used to assess potential cutoff days after vaccine and adenopathy. The analysis was done using R version 3.6.2. This study and waiver of informed consent were approved by Mayo Clinic Institutional Review Board.

## Results

Of 750 women total, 23 (3%) patients had axillary adenopathy on mammography and only 2 patients were symptomatic (Table). As summarized in the Table, presence of symptoms was associated with abnormal imaging (40% vs 60%, *P* = .01) but not age (median [range] 64 [35-83] vs 67 [31-94]; *P* = .29) and type of vaccine (*P* = .70). Most patients with adenopathy had their second vaccination prior to breast imaging (18 out of 23 patients). However, there was no significant difference between the incidence of adenopathy after the first or second vaccination (*P* = .34).

**Table.** Characteristics of Patients With and Without Adenopathy After COVID-19 Vaccination

Characteristic	No. (%)		*P* value
	Patients with adenopathy (n = 23)	Patients without adenopathy (n = 727)	
Age, median (range), y	64 (35-83)	67 (31-94)	.29
Type of imaging			.01
Diagnostic mammogram	6 (13.6)	38 (86.4)	
Screening mammogram	17 (2.4)	689 (97.6)	
Symptomatic			.01
No	21 (2.8)	724 (97.2)	
Yes	2 (40.0)	3 (60)	
Vaccine brand			.70
Moderna	14 (3.1)	432 (96.9)	
Pfizer	7 (2.4)	283 (97.6)	
Other^a^	0	5 (100)	
Unknown	2	7	
Vaccine dose			.34
First	4 (1.8)	219 (98.2)	
Second	18 (3.4)	507 (96.6)	
Unknown	1	1	
Days from vaccine, median	10 (1.0-28.0)	18.0 (1.0-85.0)	<.001
Days from vaccine			.01
1-14	15 (5.3)	268 (94.7)	
15-28	8 (2.9)	264 (97.1)	
>28	0	195 (100)	
BIRADS results			<.001
Missing	0	1	
0	15 (21.4)	55 (78.6)	
1	0	365 (100)	
2	2 (0.7)	294 (99.3)	
3	5 (45.5)	6 (54.5)	
4	0	5 (100)	
5	1 (100)	0	
6	0	1 (100)	

Abbreviation: BIRADS, Breast Imaging Reporting and Data System.

^a^ Other vaccines include Johnson & Johnson and Novavax vaccines.

The median time after vaccine in patients with adenopathy was significantly shorter at 10 days compared with 18 days in patients without adenopathy (median [range] 10 [1-28] vs 18 [1-85] days; *P* < .001). Adenopathy rates decreased as days from vaccine increased with 15 of 283 (5.3%) for 1 to 14 days, 8 of 272 (2.9%) for 15 to 28 days, and 0 of 195 (0%) for more than 28 days (*P* = .01). Using ROC analysis to identify the potential cutoff value of days after vaccination, the area under the ROC curve was 0.72 (95% CI, 0.63-0.81) with the potential cutoff of 22.5 days.

The spectrum of mammography findings ranged from a single enlarged lymph node, to multiple enlarged lymph nodes, to adenopathy with soft tissue stranding. Additional imaging with ultrasonography was requested for 21 patients. At the time of this article, 17 ultrasonography examinations had been performed. Ultrasonography findings ranged from mildly prominent nodes with a preserved fatty hilum to rounded nodes demonstrating apparent loss of a fatty hilum. Follow-up imaging recommendations included no follow-up (n = 2), repeated ultrasonography with or without mammogram in 3 months (n = 14), and biopsy (n = 1). Biopsy was recommended for a patient with an ipsilateral breast cancer. Biopsy findings for this patient were negative for malignancy, and the adenopathy was presumably vaccine induced.

## Discussion

While the incidence of COVID-19 vaccine-induced adenopathy in our study appeared to be low at 3% compared with 16% of self-reported axillary swelling in previous COVID-19 vaccine trials, this incidence is still higher than axillary adenopathy in otherwise normal mammography, which was reported as 0.02% to 0.04%.^3^ Therefore, routine inquiring about recent history of COVID-19 vaccination is warranted. The incidence of adenopathy decreased over time with no adenopathy seen in patients who received the vaccine more than 28 days previously, which supports the recommendations from Society of Breast Imaging. In addition, patients with symptomatic adenopathy are more likely to have adenopathy (odds ratio, 28.97; 95% CI, 3.23-226.09; *P* = .01). However, the present study has limitations, particularly with its small sample size and being a single center study. As COVID-19 vaccination is rolling out around the world, this study offers timing considerations and possible findings for breast imaging following vaccination. Further studies are needed to guide future recommendations for following up with patients with adenopathy after vaccination and evaluating findings with other imaging modalities.
